# Serum sclerostin as a marker of microvascular and macrovascular complications among children and adolescents with type 1 diabetes mellitus

**DOI:** 10.1007/s00467-025-06793-3

**Published:** 2025-05-12

**Authors:** Dina E. Sallam, Yasmine Ibrahim Mahmoud Elhenawy, Aya Mohamed Abdullah Ahmed, Sara Ibrahim Abdelfattah Taha, Eman Mohamed Elsayed

**Affiliations:** 1 Department of Pediatrics and Pediatric Nephrology, Faculty of Medicine, Ain Shams University, Cairo, Egypt; 2 Department of Pediatric, Faculty of Medicine, Ain Shams University, Cairo, Egypt; 3 Department of Clinical Pathology, Faculty of Medicine, Ain Shams University, Cairo, Egypt

**Keywords:** CIMT, DN, Sclerostin, T1DM

## Abstract

**Background:**

Uncontrolled diabetes mellitus (DM) accelerates atherosclerosis and vascular diseases, leading to micro- and macrovascular complications. Early cardiac and kidney involvement necessitates an early biomarker. Sclerostin is a Wnt-signaling inhibitor, having a pathophysiological role in vasculopathy, and could be used as a vasculopathy marker. Nevertheless, few data are available in pediatric patients with type 1 diabetes mellitus (T1DM). We aimed at assessing its serum level, and relation to diabetic microvascular and macrovascular complications.

**Methods:**

This is a case control study on patients with T1DM, and healthy controls. Patients were divided into non-diabetic nephropathy (DN), and DN groups according to proteinuria. Patients’ clinicodemographic and anthropometrics were obtained, with withdrawal of fasting serum lipid profile, kidney function test, and serum sclerostin. Carotid intimal media thickness (CIMT), a marker of subclinical atherosclerosis, was measured.

**Results:**

We had 75 comparable subjects, where median (IQR) serum sclerostin levels were significantly higher in DN, compared to non-DN and controls [90.83 (82.32 – 115.1), vs. 33.29 (28.37 – 38.53), vs. 13.5 (10.32 – 15.72) ng/mL,respectively, p, < 0.001]. Similarly, median (IQR) CIMT was significantly higher in DN, than in non-DN and controls [1.1 (0.8 – 1.3), vs. 0.11 (0.1 – 0.2), vs. 0.11 (0.1 – 0.2) mm, respectively, p < 0.001]. Serum sclerostin level correlated positively with disease duration, higher HgbA1c%, albuminuria level, and CIMT in all patients. The cut-off values of serum sclerostin > 60.0 ng/mL and CIMT > 0.3 mm were able to detect DN.

**Conclusions:**

Serum sclerostin levels may serve as a potential biomarker for microvascular and macrovascular complications in pediatric patients with T1DM.

**Graphical abstract:**

**Figure:**
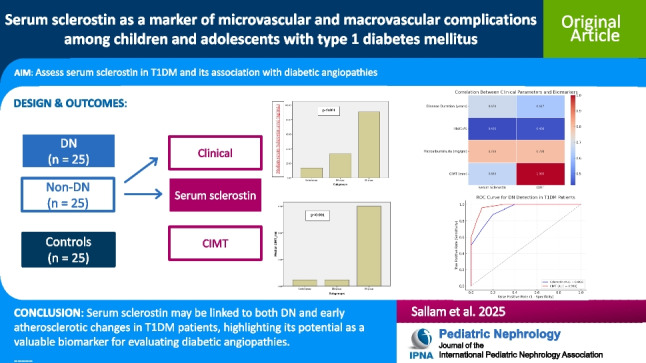
A higher resolution version of the Graphical abstract is available as Supplementary information

**Supplementary Information:**

The online version contains supplementary material available at 10.1007/s00467-025-06793-3.

## Background

Persistent hyperglycemia in uncontrolled diabetes mellitus (DM) accelerates atherosclerosis, contributing to microvascular complications such as diabetic nephropathy (DN) and macrovascular complications, including cardiovascular diseases (CVDs) [1]. Kidney involvement in diabetes manifests early, with structural, clinical, and biochemical changes that begin shortly after disease onset. These include glomerular basement membrane thickening and mesangial expansion [2], eventually progressing to albuminuria and a decline in glomerular filtration rate (GFR) [3]. If left untreated, this progression leads to chronic kidney disease (CKD), emphasizing the early onset and significance of diabetic kidney disease (DKD). CVD is a well-recognized comorbidity in patients with type 1 diabetes mellitus (T1DM), where cardiovascular remodeling occurs early after the diagnosis. Previous studies indicate that adolescents and young people with T1DM have subclinical cardiac dysfunction, central aortic stiffness, and accelerated atherosclerosis and peripheral vascular disease [4, 5]. The carotid intima-media-thickness (CIMT) measurement is a reliable surrogate marker for cardiovascular risk, enabling the early detection of atherosclerotic changes and CVDs [6].

Given the early onset of cardiac and kidney involvement in diabetes, routine microalbuminuria screening remains the clinical gold standard for diagnosing DKD in pediatric patients [7]; however, since microalbuminuria typically appears in later stages of uncontrolled diabetes, there is a critical need for earlier diagnostic biomarkers for CVDs and DN. Identifying such biomarkers could facilitate early detection, close monitoring, and timely therapeutic intervention.

Sclerostin, a key inhibitor of Wnt signaling, significantly influences both bone and vascular physiology. It contributes to vascular aging by triggering proinflammatory pathways, which facilitate intimal thickening, endothelial dysfunction, vascular smooth muscle cell (VSMC) migration and proliferation, and increased vascular calcification [8]*.* Serum sclerostin levels increase with aging and remain elevated in various pathological conditions, including DM and CKD, contributing to a higher risk of CVDs. Consequently, sclerostin has emerged as a potential biomarker for both clinical and subclinical vascular diseases [8, 9]*.* However, limited data exist regarding the association between serum sclerostin levels and diabetic angiopathy in pediatric and adolescent patients with T1DM, highlighting the need for further investigation.

Thus, the primary objective of this study was to evaluate serum sclerostin levels in children and adolescents with T1DM. Additionally, we investigated the association between sclerostin levels and diabetic angiopathies, including DN as a microvascular complication and atherosclerosis as a macrovascular complication.

## Methodology

This was a case control study that was conducted at pediatric and adolescent diabetes and nephrology units, Children’s Hospital, Faculty of Medicine, Ain Shams University, Egypt. The study was conducted after approval by the local research ethics committee at our hospital (ASU MS 658/2022), where informed assent and consent were obtained from all participants and their caregivers, respectively.

Patients diagnosed for more than three years with T1DM according to International Society for Pediatric and Adolescent Diabetes (ISPAD) guidelines [10], and between the age of 5 to 18 years and who were compliant on basal/bolus insulin regimen were recruited from the Pediatric Diabetes and Nephrology Clinic, Children’s Hospital, Faculty of Medicine, Ain Shams University, Cairo, Egypt. They were divided according to presence or absence of albuminuria into group 1: non-diabetic nephropathy patients (25 patients, non-DN), and group 2: patients with DN (25 patients), and they were age and gender comparable groups (p > 0.05). All involved T1DM patients were compared to 25 healthy age- and gender-matched controls (p > 0.05) who were recruited from the same hospital outpatient general pediatric clinic. Participants with liver dysfunction, kidney impairment, and proteinuria due to other causes than diabetes, and other autoimmune diseases, were excluded from the study.

Patients and controls were subjected to detailed history, emphasis on demographic data, duration of disease, in addition to anthropometric measurements, measurement of estimated glomerular filtration rate (eGFR) using the revised bedside Schwartz formula [11], fasting lipid profile, the mean HbA1c% over the last year prior to the study, and urinary albumin-to-creatinine ratio (UACR) in the early morning spot urine sample, which was collected at home before attending the clinic, where normal level of UACR is below 30 mg/g, and microalbuminuria between 30–300 mg/g [12]. Diabetic nephropathy was defined as having UACR ≥ 30 mg/g [12]. Serum sclerostin level was measured using ELISA technique (BT lab, Shanghai, China, cat. no E3068Hu), where standard curve range was between 0.5–200 ng/ml, with sensitivity of 0.26 ng/ml [13]. The CIMT as an early marker for atherosclerosis, was determined in accordance with the Mannheim Consensus Guidelines 2011 [14] by a skilled radiologist who was unaware of the patient’s clinical status. The ultrasound evaluation was conducted using a single device at our Children’s Hospital that was equipped with a linear high-frequency transducer operating with a 7–10.0-MHz linear array. The examination took place after a 10-min rest period, with the patient lying in a supine position and their neck slightly extended, while their head was turned slightly towards the opposite side. Measurements were captured from the far wall of the common carotid artery on both sides, approximately 10 to 20 mm proximal to the bifurcation point. The thickest CIMT measurements were determined using calipers on a magnified image of the common carotid artery. These measurements were taken at the end of diastole. The result was calculated as the average of three measurements taken on each side.

## Statistical analysis

The collected data was revised, coded, and tabulated using statistical package for Social Science (IBM Corp. Released 2017. IBM SPSS Statistics for Windows, Version 25.0. Armonk, NY: IBM Corp.). Data were presented and suitable analysis was done according to the type of data obtained for each parameter, where Shapiro–Wilk test was done to test the normality of data distribution, and descriptive statistics were analyzed using mean and standard deviation for parametric data, median and range for non-parametric data. Analytical statistics were performed using Student’s t test to assess the statistical significance of the difference of parametric variable between two study group means, Mann–Whitney test (U test) to assess the statistical significance of the difference of a non-parametric variable between two study groups, while Kruskal–Wallis test was used to assess the statistical significance of the difference of a non-parametric variable between more than two study groups, and Chi-Square test was used to examine the relationship between two qualitative variables, in addition to the correlation analysis which was done to assess the strength of association between two quantitative variables. The ROC curve (receiver operating characteristic) provided a useful way to evaluate the sensitivity and specificity for quantitative diagnostic. We performed a ROC analysis to evaluate the diagnostic accuracy of serum sclerostin and CIMT in detecting diabetic nephropathy (DN) among children and adolescents with T1DM (groups 1 and 2), where healthy controls were not included in the ROC analysis. The optimum cut-off point was defined as that which maximized the AUC value, where AUC with an area greater than 0.9 has high accuracy, while 0.7–0.9 indicates moderate accuracy, 0.5–0.7, low accuracy and 0.5 a chance result.

## Results

Clinical and demographic data of our studied cohort are illustrated in Table 1. The current study included 75 participants divided equally into 3 groups: group 1 (non-DN patients), group 2 (DN), and healthy controls with mean ± SD age of 11.18 ± 3.16, 12.08 ± 2.29, and 10.80 ± 4.23 years, respectively, where there was no statistically significant difference between them regarding age, gender, and anthropometric measure (p > 0.05). In all of our patients, both systolic and diastolic blood pressure were normally below the 90 th percentile, considering the patient's sex, age, and height. There was a significant difference between patients of group 1 and group 2 regarding the disease duration, where the patients with DN had longer duration of diabetes [median (IQR) = 8 (6–11), vs. 4 (3–5) years, respectively, p < 0.001]. As anticipated, patients with DN had higher levels of UACR (64.33 ± 13.45 vs. 9.87 ± 3.21, p < 0.001) and they had significantly higher levels of HbA1c% (11.13 ± 1.91 vs. 10 ± 1.27%, p = 0.018), meanwhile, fasting lipid profile and eGFR was comparable between the T1DM groups (Table 2).

**Table 1:** Clinic-demographic and laboratory data of the studied cohort

All subjects		Controls n = 25	Group 1 (DM, non-DN) n = 25	Group 2 (DN) n = 25	p-value
Age (years)	Mean ± SD	10.80 ± 4.23	11.18 ± 3.16	12.08 ± 2.29	0.381
	Range	7–14	7–15	7–17	
Gender (n, %)	Female	13 (52.0%)	16 (64.0%)	18 (72.0%)	0.339
	Male	12 (48.0%)	9 (36.0%)	7 (28.0%)	
BMI SDS	Median (IQR)	0.55 (0.4–1)	0.48(0.11–1)	0.53(0.3–0.9)	0.305
Duration of diabetes (years)	Median (IQR)		4 (3–5)	8 (6–11)	0.000
Serum creatinine (mg/dL)	Mean ± SD	0.37 ± 0.12	0.41 ± 0.15	0.39 ± 0.19	0.73
	Range	0.1–0.6	0.2–0.7	0.2–0.7	
eGFR (ml/min/1.73 m^2^)	Mean ± SD		108.31 ± 6.82	109.7 ± 5.32	0.4223
HbA1c%	Mean ± SD		10 ± 1.27	11.13 ± 1.91	0.018
Total serum cholesterol (mg/dL)	Median (IQR)		100 (50–149)	86 (60–160)	0.946
Serum triglycerides (mg/dL)	Median (IQR)		82 (65–113)	110 (66–124)	0.228
UACR (mg/g creatinine)	Mean ± SD		9.87 ± 3.21	64.33 ± 13.45	< 0.001
Serum sclerostin (ng/mL)	Median (IQR)	13.5 (10.32–15.72)	33.29 (28.37–38.53)	90.83 (82.32–115.1)	< 0.001
	Range	7.03–19.33	17.23–60.01	43.63–145.8	
CIMT (mm)	Median (IQR)	0.11 (0.1–0.2)	0.11 (0.1–0.2)	1.1 (0.8–1.3)	< 0.001
	Range	0.1–0.3	0.01–0.3	0.3–2	

*BMI*, body mass index; *CIMT*, carotid intima media thickness; *DM*, diabetes mellitus; *DN*, diabetic nephropathy; *eGFR*, estimated glomerular filtration ratio; *HBA1c*, hemoglobin A1c; *SD*, standard deviation; *UACR*, urinary albumin creatinine ratio

**Table 2:** Correlation of serum sclerostin and CIMT with other studied parameters among all participants with T1DM

All T1DM patients (n = 50)	Serum sclerostin		CIMT	
	r	p-value	r	p-value
Age (years)	0.132	0.371	0.034	0.784
Disease duration (years)	0.678	< 0.001	0.617	< 0.001
HbA1c%	0.426	0.002	0.436	0.002
Microalbuminuria (mg/gm)	0.798	< 0.001	0.798	< 0.001
Cholesterol (mg/dL)	0.022	0.881	−0.022	0.878
Triglycerides (mg/dL)	0.155	0.284	0.114	0.432
Serum Sclerostin (ng/mL)	-	-	0.66	< 0.001
CIMT (mm)	0.66	< 0.001	-	-

*CIMT*, carotid intima media thickness; *DM*, diabetes mellitus; *HBA1c%,* hemoglobin A1c%; r, Spearman correlation coefficient

**Fig. 1:**
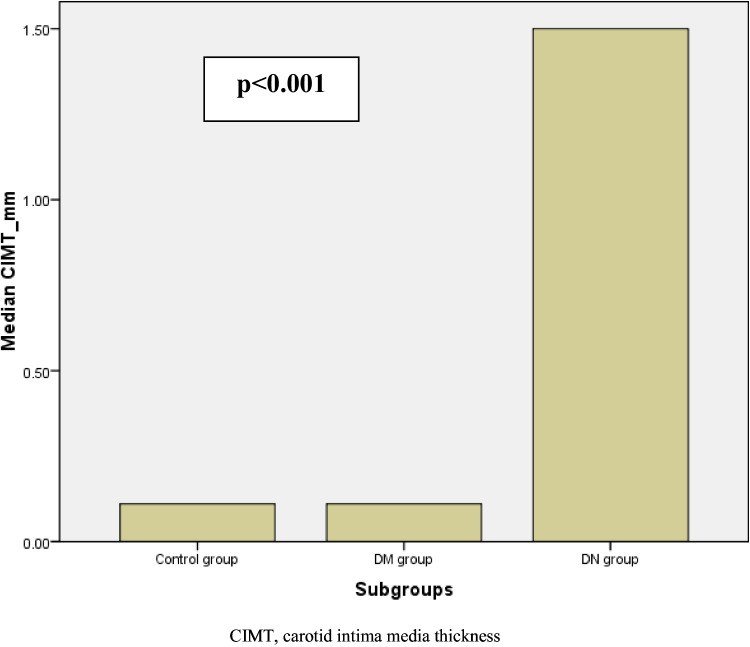
CIMT (mm) among the studied cohort. CIMT, carotid intima media thickness

Serum sclerostin level was significantly higher in all T1DM patients compared to healthy controls while the median serum sclerostin level was significantly higher among patients with DN [90.83 (82.32–115.1) vs. 33.29 (28.37–38.53), p < 0.001] (Table 1 and Fig. 1).

**Fig. 2:**
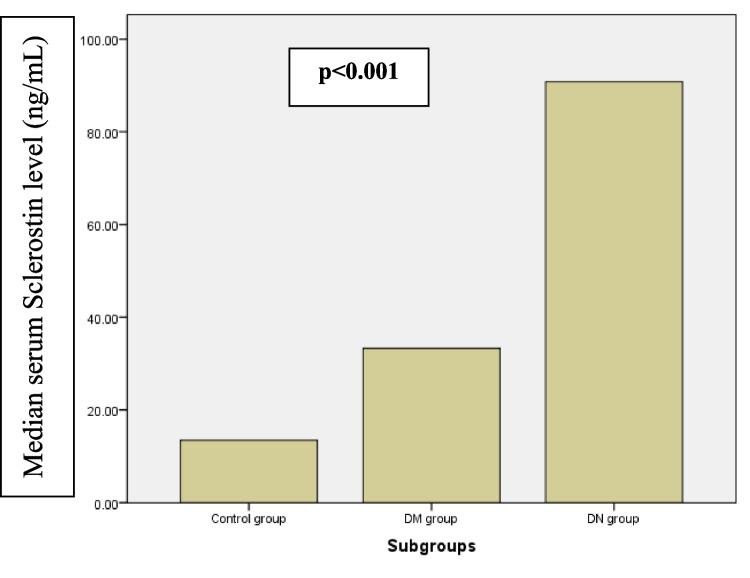
Serum sclerostin levels (ng/mL) among the studied cohort

Although CIMT wasn’t changed significantly between the control and non-DN groups (p = 0.7), it was significantly higher in DN than non-DN patients and controls, which was evident by post hoc analysis [control 0.11 (0.1–0.2) vs. non-DN 0.11 (0.1–0.2) vs. DN 1.1 (0.8–1.3) ng/mL), p < 0.001] (Table 1 and Fig. 2).

**Fig. 3:**
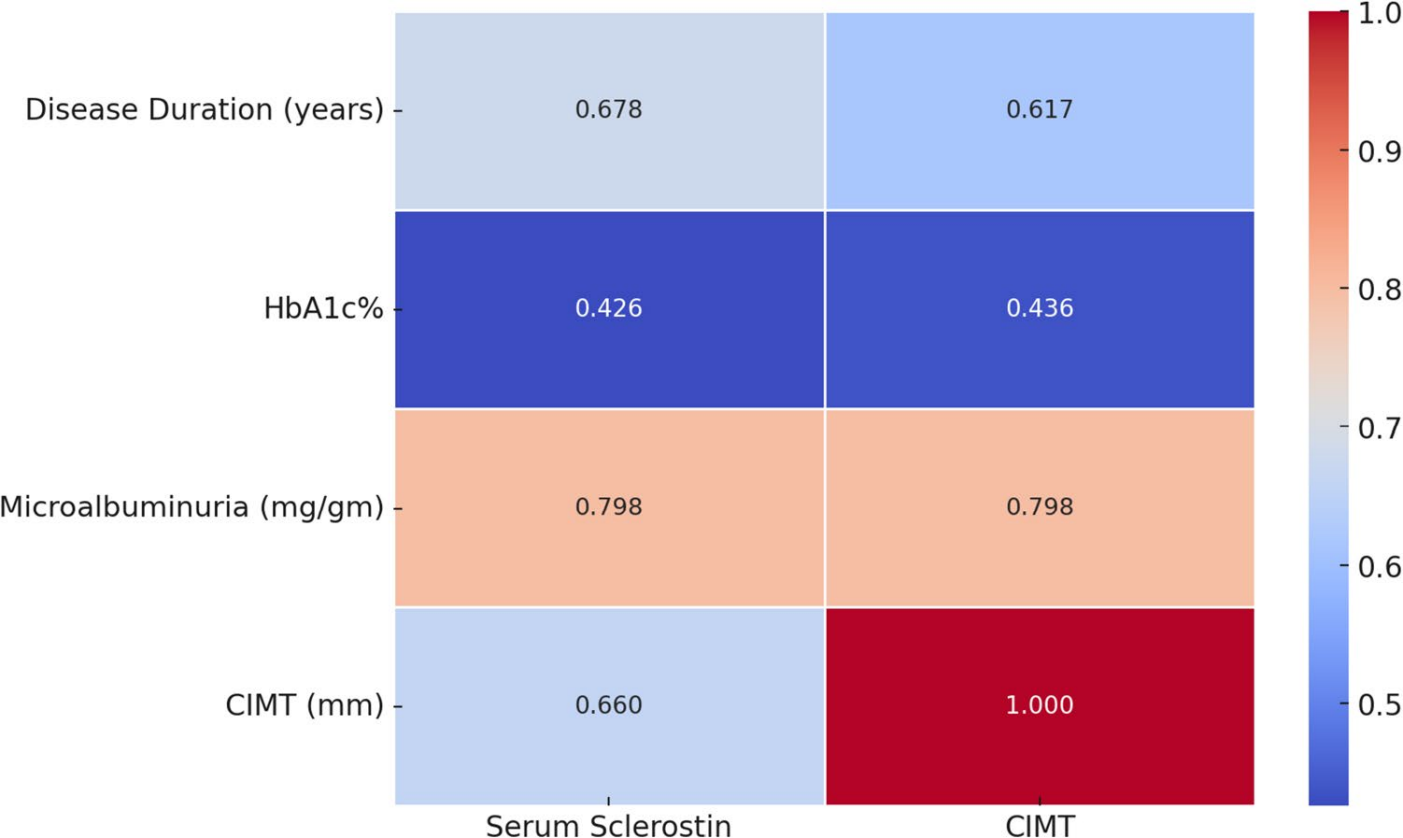
Heatmap correlations between clinical parameters, CIMT and serum sclerostin levels in the DN group

**Fig. 4:**
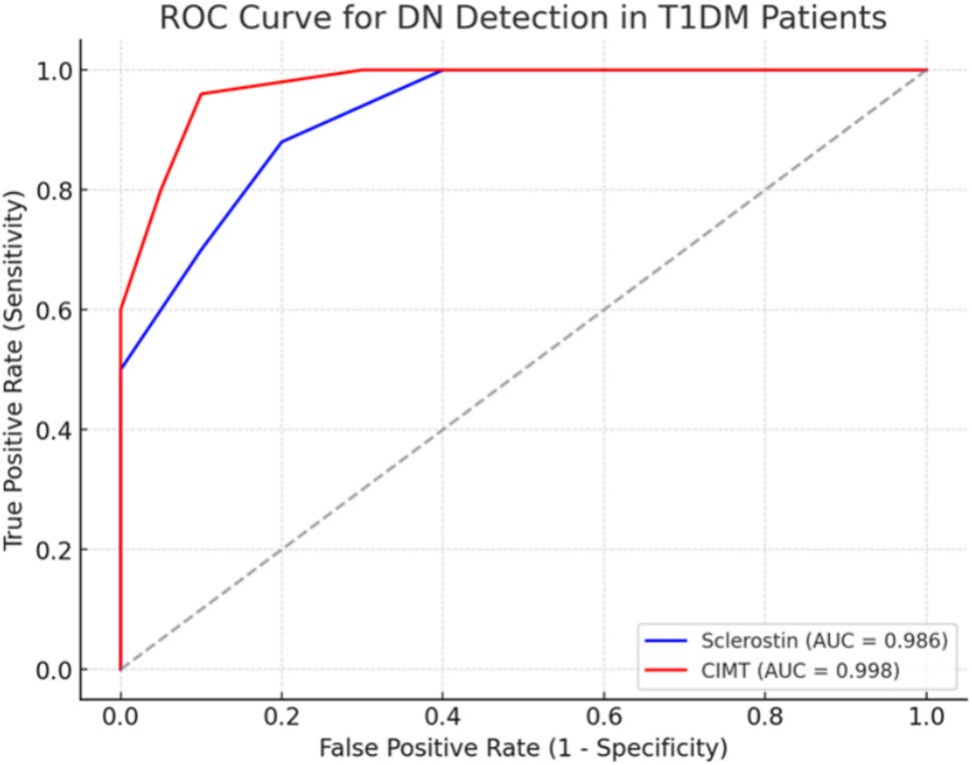
ROC curve of serum sclerostin and CIMT in DN. CIMT, carotid intima media thickness; DN, diabetic nephropathy

Among participants with T1DM, serum sclerostin level positively correlated with the disease duration, HbA1c%, UACR, and CIMT (r = 0.678, 0.426, 0.798, 0.66, respectively, p < 0.005) (Table 2). Also, in the DN group, serum sclerostin level significantly positively correlated with the duration of diabetes, HbA1 C, and UACR, and CIMT (r = 0.678, 0.426, 0.798, 0.66, respectively, p < 0.005) (Table 3, Fig. 3). We have conducted the ROC analysis to assess the diagnostic performance of both serum sclerostin and CIMT in detecting DN in children and adolescents with T1DM (n = 50), divided into those with DN (n = 25) and without DN (n = 25), where healthy controls were not included. The ROC analysis demonstrates that both serum sclerostin and CIMT are highly accurate markers for detecting DN in children and adolescents with T1DM, where it identified an optimal cut-off value of 60.01 ng/mL for serum sclerostin (AUC = 0.986, sensitivity = 88%, and specificity = 100%) and a cut-off value of 0.3 mm for CIMT (AUC = 0.998, sensitivity = 96%, and specificity = 100%) (Fig. 4).

**Table 3:** Correlation of serum sclerostin and CIMT with other studied parameters among participants with DN

DN group	Sclerostin factor		CIMT	
	r	P-value	r	P-value
Age (years)	0.176	0.400	0.174	0.405
Duration(years)	0.507	0.004	0.469	0.005
HbA1c%	0.688	< 0.001	0.753	< 0.001
Microalbuminuria	0.798	0.000	0.617	0.001
Cholesterol(mg/dL)	0.089	0.672	0.154	0.461
Triglycerides (mg/dL)	0.060	0.775	0.055	0.796
CIMT (mm)	0.512	0.009		
Serum Sclerostin level (ng/mL)			0.512	0.009

*CIMT*, carotid intima media thickness; *DN*, diabetic nephropathy; *HBA1c*, hemoglobin A1c; *TG*, triglyceride

P-value > 0.05, non-significant; P-value < 0.05, significant; P-value < 0.01, highly significant; r, Spearman correlation coefficient

## Discussion

In our study, serum sclerostin level was significantly higher in patients with diabetes than in controls, especially in the DN group, which indicates an association of serum sclerostin level with glucose metabolism. Mechanisms involved in development and progression of DN are still unclear, however, the oxidative stress and vascular changes are incriminated and linked directly to DN pathophysiology. Also, it is known that sclerostin has a known role in vascular pathology and increased oxidative stress, especially in diabetic patients [8, 9], and a possible role in the development of DN.

Very few previous studies on serum sclerostin levels in pediatric and adolescent patients with T1DM are available, and the findings remain controversial. In accordance with our results, a previous Japanese study [15] that involved 22 patients with T1DM with mean (± SD) age of 14.2 ± 4.2 years and 16 age-, sex- and sex hormone-matched healthy controls, where they found significantly elevated serum sclerostin levels in patients with T1DM than in controls, without significant differences between genders. The study found a significant positive association with height and body mass index standard deviation, and bone-specific alkaline phosphatase, which suggested the role of sclerostin in bone fragility in Japanese children and adolescents with T1DM, and a potential association between sclerostin and glucose metabolism. Additionally, a previous Italian pediatric cross-sectional study [16] reported similar findings, where they involved 106 patients with T1DM with mean ± SD age of 12.2 ± 4 years, and age- and gender-matched 80 controls, where significantly increased serum levels of sclerostin were found in patients with T1DM with respect to controls; the study investigators explained their findings due to the fact that sclerostin plays a crucial role in regulating bone mass, and individuals with T1DM exhibit low bone turnover, primarily due to impaired bone formation. Consequently, these patients tend to have elevated levels of sclerostin, a Wnt-signaling inhibitor. Meanwhile a Greek study [17] on 40 children and adolescents with T1DM with mean (± SD) age and T1DM duration of 13.04 ± 3.53 and 5.15 ± 3.33 years, respectively, and 40 healthy age- and gender-matched controls, found no differences in serum sclerostin levels between T1DM patients and their healthy peers.

In our study, serum sclerostin positively correlated with HbA1c% and glycemic control, where higher serum sclerostin was associated with bad glycemic control. On the contrary, an Italian pediatric study [16] found that serum sclerostin levels improved in individuals with better glycemic control, suggesting that measuring serum sclerostin could serve as a useful tool for simultaneously monitoring glycemic status in T1DM patients. Furthermore, multiple regression analyses indicated that glycemic control was the strongest predictor of sclerostin levels. Additionally, a Polish study [18] involving 55 obese children and adolescents with a mean age of 13.2 ± 3.4 years, and 26 sex- and Tanner stage-matched healthy controls, examined the relationship between serum sclerostin levels and insulin resistance. The study found a negative correlation between sclerostin and HOMA-IR and insulin levels in obese patients, while in healthy individuals, sclerostin was negatively correlated only with C-peptide. No correlation was observed between sclerostin and HbA1c in any group. The researchers concluded that sclerostin may play a significant role in regulating glucose metabolism in children and adolescents, independent of other fat- and bone-derived factors, and that in obese youth, it may contribute to reducing insulin resistance.

We found that serum sclerostin levels in all of our T1DM patients didn’t correlate with gender, which was in accordance with a previous Japanese study [15], while previous Italian [16] and Greek [17] pediatric studies contrasted with our findings, where they found that gender influenced sclerostin levels, with higher levels observed in male T1DM patients compared to females. Additionally, sclerostin levels correlated with pubertal stage, which the researchers attributed to the influence of sex hormones on sclerostin regulation.

We found that serum sclerostin levels in all of our T1DM patients correlated positively with disease duration, where longer disease duration was associated with higher serum sclerostin level. Similarly, a previous study [19] on adult patients with T2DM found a similar positive correlation between serum sclerostin levels and disease duration. The researchers hypothesized that Wnt signaling pathway dysregulation in T2DM may become more pronounced with longer disease duration.

We used CIMT as a marker for subclinical atherosclerosis, where it was significantly elevated in patients with diabetes with DN, than in non-DN patients and controls, which was confirmed by post hoc analysis. This confirms that patients with DN have a risk of development of atherosclerosis and hence macrovascular complications, owing to many mechanisms including endothelial dysfunction, and glycocalyx disruption, which explain why patients with diabetes with albuminuria have a notably greater cardiovascular event risk than diabetics with normoalbuminuria [20, 21]. Additionally, in our study we found that CIMT correlated positively with serum sclerostin level in all T1DM patients, highlighted in the DN group who are at high risk of diabetic macroangiopathy. Our results confirm the previous studies [22, 23], where they had shown that sclerostin was expressed and detected in the VSMCs of atherosclerotic plaques, and in the aorta of patients undergoing aortic valve replacement and is elevated in VSMCs and calcified valvular plaques, pointing to its role in atherosclerosis development.

The diagnostic performance of serum sclerostin and CIMT in detecting DN among pediatric and adolescent patients with T1DM was evaluated using ROC curve analysis. The results indicate that both markers could serve as reliable and non-invasive biomarkers for the early identification of DN, potentially before the onset of overt kidney impairment. Notably, CIMT demonstrated slightly superior diagnostic accuracy. The high AUC values, coupled with excellent sensitivity and specificity for both markers, underscore their potential clinical utility in the early detection and risk stratification of DN in this population. To our knowledge, no previous publication has studied the cut-off level of serum sclerostin and CIMT to detect and diagnose DN, hence we recommend further studies on a wider scale to study the sensitivity of these markers as early detectors of DN.

Our study is one of a kind owing to measuring the serum sclerostin levels in an Egyptian homogeneous cohort of pediatric and adolescent patients with T1DM with and without DN, in comparison to healthy controls. Additionally, this work presents new information that may point to sclerostin's involvement in T1DM micro- and macrovascular complications. A limitation of our study is that we did not perform a comprehensive cardiac assessment using echocardiography; instead, we focused solely on measuring blood pressure and CIMT. Additionally, we did not evaluate other laboratory parameters, such as parathyroid hormone (PTH) and alkaline phosphatase (ALP). Consequently, we were unable to analyze their correlation with serum sclerostin levels and proteinuria. Finally, other limitations of our study are the inclusion of a relatively small sample size and it being a single-center design.

## Conclusions

Our study demonstrated that serum sclerostin levels were significantly elevated in patients with T1DM, particularly in those with DN. Additionally, CIMT was significantly higher in DN patients, suggesting an association with vascular changes. Serum sclerostin correlated positively with disease duration, HbA1c%, UACR, and CIMT, highlighting its potential role in DN pathophysiology. Receiver operating characteristic (ROC) analysis identified serum sclerostin and CIMT as highly sensitive and specific biomarkers for DN detection in T1DM patients. These findings indicate that serum sclerostin may be linked to both DN and early atherosclerotic changes in patients with T1DM, highlighting its potential as a valuable biomarker for evaluating diabetic angiopathies.

## Supplementary Information

Below is the link to the electronic supplementary material.

Graphical abstract (PPTX 244 KB)

## Data Availability

Data are available upon request.

## Declarations

This manuscript has been posted on a nonprofit preprint server.
