# Supplementary material for: Serum sclerostin as a marker of microvascular and macrovascular complications among children and adolescents with type 1 diabetes mellitus
Source: Pediatr Nephrol. 2025 May 12;40(10):3155–62. doi: 10.1007/s00467-025-06793-3 (PMC12402022; doi:10.1007/s00467-025-06793-3)
Supplement: Supplementary file 1 — Graphical abstract (PPTX 244 KB) [file 467_2025_6793_MOESM1_ESM.pptx]

## Slide 1
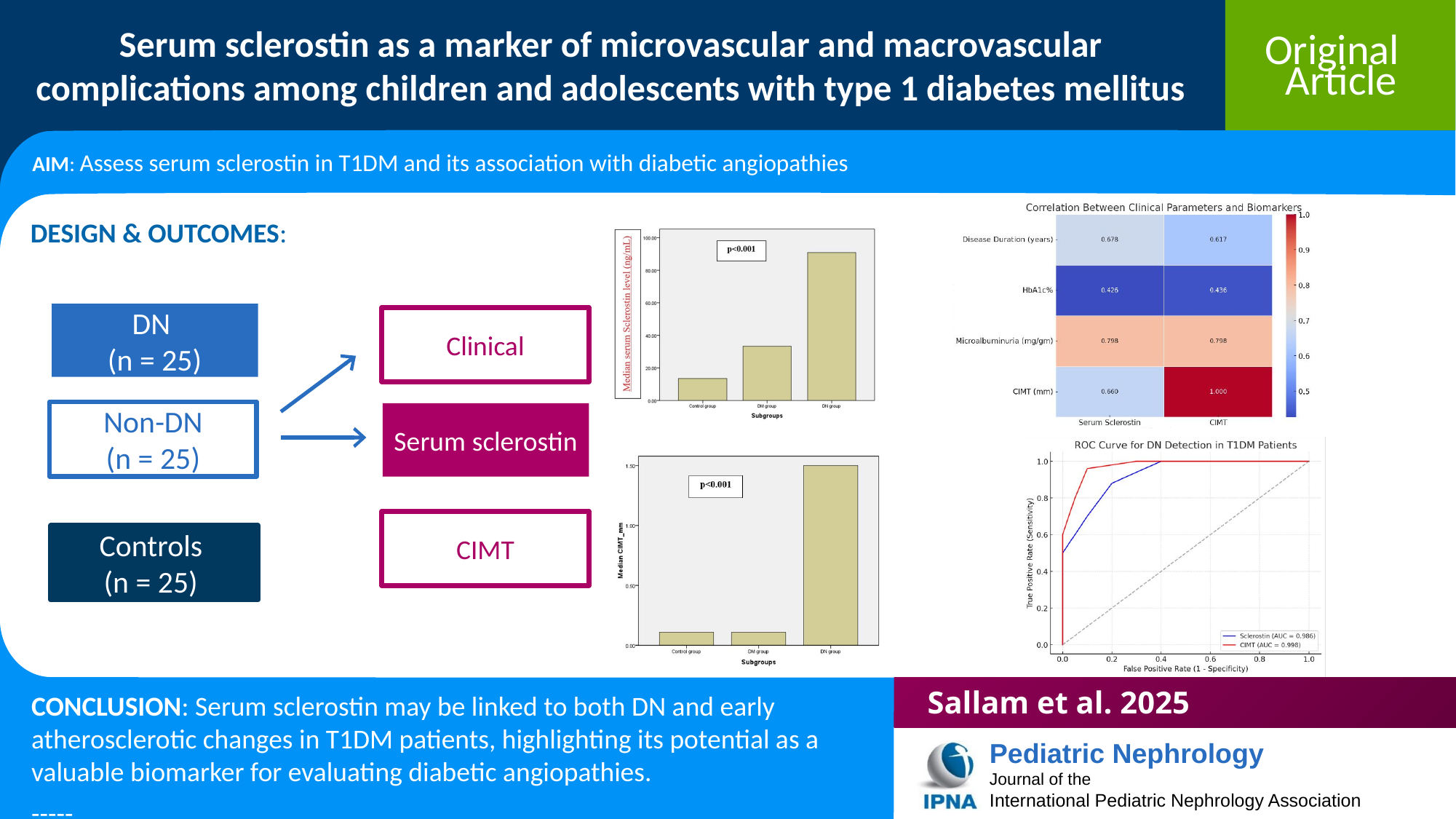

Serum sclerostin as a marker of microvascular and macrovascular complications among children and adolescents with type 1 diabetes mellitus
AIM: Assess serum sclerostin in T1DM and its association with diabetic angiopathies
DESIGN & OUTCOMES:
DN
(n = 25)
Clinical
Non-DN
(n = 25)
Serum sclerostin
CIMT
Controls
(n = 25)
Sallam et al. 2025
CONCLUSION: Serum sclerostin may be linked to both DN and early atherosclerotic changes in T1DM patients, highlighting its potential as a valuable biomarker for evaluating diabetic angiopathies.
-----
